# Nature of ammonia storage sites in H-SSZ-13 and Cu-SSZ-13

**DOI:** 10.1039/d6ra03025d

**Published:** 2026-05-26

**Authors:** Ghodsieh Isapour, Yingxin Feng, Henrik Grönbeck, Magnus Skoglundh, Hanna Härelind

**Affiliations:** a Competence Centre for Catalysis, Chalmers University of Technology Gothenburg Sweden; b Department of Chemistry and Chemical Engineering, Division of Applied Chemistry Sweden hanna.harelind@chalmers.se ghodsieh@chalmers.se; c Department of Physics, Division of Chemical Physics Sweden

## Abstract

In this study, the nature of ammonia storage sites in H-SSZ-13 and Cu-SSZ-13 with different Si/Al and Cu/Al molar ratios is characterized by NH_3_ sorption experiments using *in situ* DRIFT spectroscopy, and by NH_3_-TPD flow reactor experiments. In addition, the binding energy and vibrational properties of different surface species are obtained by DFT calculations to aid the interpretation of the experimental results. The NH_3_ sorption DRIFTS experiments over Cu-free samples show that NH_3_, NH_4_^+^ and NH_4_^+^·NH_3_ ions form during the adsorption of NH_3_ over the Brønsted acid sites at 70 °C. DRIFT results show that peaks at around 3735 and 3650 cm^−1^ become increasingly more negative with increasing Cu loading, which is likely related to solvation of Cu by NH_3_. DFT calculations show two possible ammonia-solvated Cu species, *i.e.* Cu^I^(NH_3_)_2_ and Cu^II^(NH_3_)_3_OH. By comparing the simulated and measured IR spectra, we conclude that the experiments match the simulated infrared spectra of Cu^II^(OH)(NH_3_)_3_, Cu^I^(NH_3_)_2_, NH_4_^+^ and NH_4_^+^·NH_3_. Both DRIFTS experiments and calculations show that the amounts of Cu^I^(NH_3_)_2_ and Cu^II^(OH)(NH_3_)_3_ increase with decreasing Si/Al molar ratio and increasing Cu loading. Furthermore, the TPD results show that the NH_4_^+^·NH_3_ peak decomposes at relatively low temperatures, *i.e.* in the low-temperature region. The remaining NH_4_^+^ species is stable until decomposition in the high-temperature region.

## Abstract 2

In this study, the nature of ammonia storage sites in H-SSZ-13 and Cu-SSZ-13 with different Si/Al and Cu/Al molar ratios is characterized by NH_3_ sorption experiments using *in situ* DRIFT spectroscopy, and by NH_3_-TPD flow reactor experiments.

## Graphical abstract

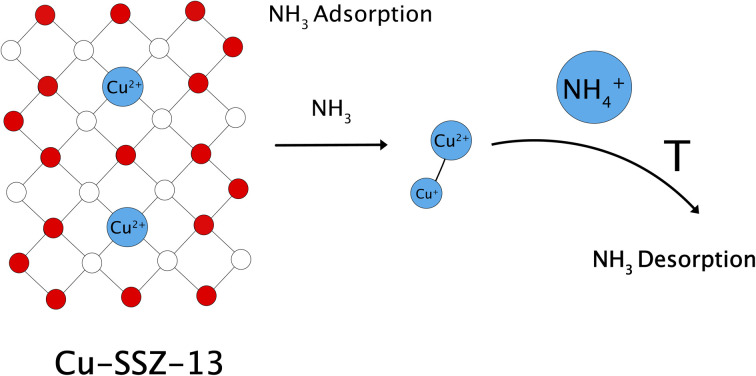

## 1. Introduction

The development of catalysts for NO_*x*_ abatement from (bio)diesel and hydrogen combustion is important to meet increasingly stringent regulations, not the least for heavy-duty and shipping applications. Small-pore zeolites with the chabazite framework structure (CHA), such as Cu-SSZ-13, are efficient for selective catalytic reduction of NO_*x*_ using ammonia (NH_3_-SCR) thanks to their high activity, N_2_ selectivity and hydrothermal stability in comparison with medium- and large-pore zeolites.^5,9,31,32,37,38,43^ Ammonia has different roles in the NH_3_-SCR reaction over Cu-based zeolites. Ammonia is a reactant, it solvates Cu species at temperatures below 250 °C, and it forms ammonium over Brønsted acid sites. Moreover, ammonia has been suggested to hinder the NH_3_-SCR reaction.^11^ Thus, characterization of ammonia species in Cu-SSZ-13 is important to develop understanding of the catalyst material.

To characterize the number and strength of acid sites in the zeolite, ammonia temperature-programmed desorption (NH_3_-TPD) is widely used.^15,19,25,34^ The method is straightforward and is based on the measurement of the NH_3_ desorption profile of pre-adsorbed NH_3_ during controlled temperature.^6^ In spite of its simplicity, the interpretation of TPD profiles can be challenging and hindered mainly by limited knowledge of the sample heterogeneity and its ammonia storage sites.^6,32^ Hence, due to the inherent complexities, NH_3_-TPD is generally used as a signature for the acidity of the catalyst rather than to obtain specific information regarding various sites.

In the case of NH_3_-TPD profiles for copper functionalized zeolites in flow reactor studies, three desorption peaks are typically observed;^10,35^ one peak at low temperature below 200 °C, an intermediate-temperature peak at around 250–350 °C, and a high-temperature peak at around 400–500 °C.^6,32^ Although there is no common agreement for the assignment of these peaks, it is mainly believed that the low-temperature peak is owing to weakly bonded NH_3_ on weak Lewis acid sites (Al Lewis acid sites), the medium-temperature peak is assigned to NH_3_ adsorbed either on isolated Cu^+^ or Cu^2+^ ions (strong Lewis acid sites)^7,33^ or both Lewis and Brønsted acid sites,^36^ and the high-temperature peak is attributed to NH_3_ desorption from Brønsted acid sites.^7,32,33^ However, calculations by Chen *et al.*^6^ suggest that these three experimentally observed desorption peaks could be assigned to several overlapping features. They conclude that the low-temperature peak originates from Lewis acid sites and also from decomposition of [Cu^II^(OH)(NH_3_)_3_]^+^ species. The decomposition of [Cu^I^(NH_3_)_2_]^+^ species and a residual from [Cu^II^(OH)(NH_3_)_3_]^+^ could contribute to the intermediate-temperature peak. The largest overlap seems to be for the high-temperature peak originating from decomposition of species such as NH_4_^+^, [Cu^I^NH_3_]^+^, and [Cu^II^(NH_3_)_4_]^2+^. One complication in the interpretation of NH_3_-TPD profiles is the dynamic character of the catalyst. The species present in the catalyst are highly sensitive to the pre-treatment of the NH_3_-TPD profiles, complementary experimental and theoretical techniques, such as diffuse reflectance infrared Fourier transform spectroscopy (DRIFTS) and density functional theory (DFT) may be used.

While previous studies have investigated NH_3_ storage in Cu-SSZ-13, a consistent molecular-level correlation between DRIFTS features, NH_3_-TPD profiles, and DFT-derived structures across simultaneous variations in both Si/Al ratio and Cu loading remains lacking. In this work, we combine *in situ* DRIFTS, NH_3_-TPD, and DFT calculations to establish a direct link between spectroscopic signatures, desorption behavior, and specific NH_3_-containing species. This approach enables a more comprehensive understanding of how framework composition and Cu content jointly govern ammonia storage mechanisms.

## 2. Experimental

### 2.1. Catalyst preparation and characterization

Na-SSZ-13 samples with *n*(Si)/*n*(Al) = 12, and 24, were synthesized *via* sol–gel and hydrothermal crystallization. The synthesis of these samples is according to the procedure described in detail previously. The zeolite sample with *n*(Si)/*n*(Al) = 6 was synthesized using a procedure similar to that reported by Fickel *et al.*^12,13^ Sodium silicate solution (Sigma-Aldrich, reagent grade) and NaOH (Fisher Scientific) were mixed in water and stirred for 15 min at room temperature. Then, NH_4_-Y (Zeolyst CBV100) was added to the previous solution and stirred for 30 min. Subsequently, the final solution was transferred into Teflon-lined autoclaves and heated at 140 °C under stirring for 6 days. The product was recovered by centrifuging and washed with Milli-Q water and then dried at room temperature. The as-made powder was then calcined in air at 550 °C for 8 h. All zeolite samples in H-form were Cu-exchanged by incipient wetness impregnation to obtain samples with a Cu loading corresponding to *n*(Cu)/*n*(Al) = 0.1, 0.2, 0.3, and 0.4. The precursor of Cu was Cu(NO_3_)_2_·2.5H_2_O. After impregnation and drying samples overnight at room temperature, the samples were calcined in air at 600 °C for 6 h and thereafter at 750 °C for 2 h, with a heating rate of 2 °C min^−1^. For the sample with Si/Al = 6, the calcination temperature was 450 °C for 5 h.

N_2_ physical adsorption–desorption isotherm experiments were conducted at 77 K using a Tristar 3000 (Micromeritics) instrument. The specific surface area and pore volume was calculated by the Brunauer–Emmett–Teller (BET) multiple points and Barrett–Joyner–Halenda (BJH) methods, respectively. Prior to the experiments, all fresh powder samples were degassed at 220 °C for 10 h under the flow of N_2_. The crystal structures of the samples were determined by powder X-ray diffraction (XRD) using a Bruker Siemens diffractometer D5000 operating at 40 kV and 40 mA with Cu Kα X-ray source (*λ* = 1.5418 Å). The diffraction patterns were obtained in a diffraction angle ranging from 2*θ* = 5 to 60° applying a step size of 0.017° and 1 s per step. The surface morphologies of the sample crystallites were investigated by scanning electron microscopy (SEM), using a Zeiss Ultra 55 FEG instrument equipped with an energy-dispersive X-ray (EDX) system (Oxford Inca). The elemental contents of the samples were acquired by inductively coupled plasma sector field mass spectrometry (ICP-SFMS) by ALS Scandinavia AB in Luleå, Sweden.

### 2.2. Ammonia sorption experiments

To investigate the interaction between NH_3_ and the catalyst, the nature, *i.e.,* number and strength, of the acid sites was characterized by NH_3_ adsorption–desorption experiments using DRIFT spectroscopy. The samples were pretreated first at 300 °C in Ar and 10% O_2_, and then at 250, 200, and 150 °C with 400 ppm NH_3_ and 400 ppm NO for 30 min at each temperature. This treatment gives rise to predominantly [Cu^I^(NH_3_)_2_]^+^ complexes, which has been previously shown by *in situ* X-ray absorption spectroscopy.^24^ This procedure was followed by an Ar flow at 320 °C for 2 h to decompose the Cu–ammine complex leaving framework bound Cu^I^. Background spectra were taken under Ar at the subsequent adsorption–desorption temperatures. The experiment started with an isothermal NH_3_ adsorption phase at 70 °C, where the sample was exposed to 400 ppm of NH_3_ in Ar, until saturation. This was followed by an NH_3_ desorption phase in Ar where the temperature was increased stepwise to 200 °C. The IR spectra were recorded using a VERTEX70 spectrometer (Bruker), equipped with a liquid nitrogen-cooled mercury cadmium telluride (MCT) detector (bandwidth of 600–12000 cm^−1^), and a high-temperature stainless steel reaction chamber (Harrick Scientific Products, Inc.) with CaF_2_ windows. All spectra measurements were recorded between 400 and 4000 cm^−1^ with a spectral resolution of 1 cm^−1^. The gas flow was controlled by individual mass flow controllers (HiTech) before introduced to the chamber, with a total flow of 100 ml min^−1^ (balanced with Ar).

Temperature programmed desorption of ammonia was done using powder samples in a flow reactor.^20^ A total flow of 20 ml min^−1^ was used for all experiments, with Ar as balance. The pretreatment of the samples was the same as used for the DRIFTS studies. The NH_3_-TPD experiments were carried out first by exposing the sample to 400 ppm NH_3_ in Ar at 70 °C for 30 min, purging with Ar, and finally by linearly increasing the temperature to 500 °C in Ar using a heating rate of 10 °C min^−1^. The outlet gas composition was continuously analysed by mass spectrometry (Hiden Analytical, HPS-20 QIC), following the *m*/*z* ratios 17 (NH_3_), and 40 (Ar).

### 2.3. DFT calculations

Spin-polarized density functional theory calculations were carried out using the Vienna *ab initio* simulation package (VASP).^28–30^ The interaction between the valence electrons and the core electrons was described with the projector augmented wave (PAW)^2,26^ method and the Kohn–Sham orbitals were expanded with a plane wave basis with a cutoff energy of 480 eV. The number of valence electrons treated in the calculations was Cu(11), Si(4), Al(3), O(6), N(5), and H(1). The *k*-point sampling was restricted to the gamma point. Exchange–correlation effects were described using the gradient-corrected Perdew–Burke–Ernzerhof (PBE) functional.^41^ A Hubbard-U term was introduced (PBE + *U*) to describe the highly localized Cu 3d-electrons. The *U*-value of Cu 3d was set to be 6 eV.^23^ Moreover, a Grimme-D3 correction^18^ was added to describe van der Waals interactions that should be accounted for in zeolite chemistry. The convergence criterion of self-consistent-field cycles was set to 1 × 10^−5^ eV and the structures were relaxed until the force on each atom was lower than 0.02 eV Å^−1^. Optimized structures were used for frequency analyses and the Zenodo program was employed to obtain the infrared intensities.^8^ We used a rhombohedral unit cell to describe the chabazite structure that included 12 tetrahedral Si-sites with lattice parameters that were fixed to the experimentally determined values (*α* = *β* = *γ* = 94.2 Å, *a* = *b* = *c* = 9.42 Å). To obtain CHA with different Si/Al and Cu/Al molar ratios, one or two Si atoms in the six-membered ring of the large zeolite cage were substituted by Al giving Si/Al ratios of 11 or 5. The chosen Si/Al ratios are within the experimental range^14,15,40^ and consistent with the samples synthesized in this work.

## 3. Results and discussion

### 3.1. Catalyst characterization

**Fig. 1:**
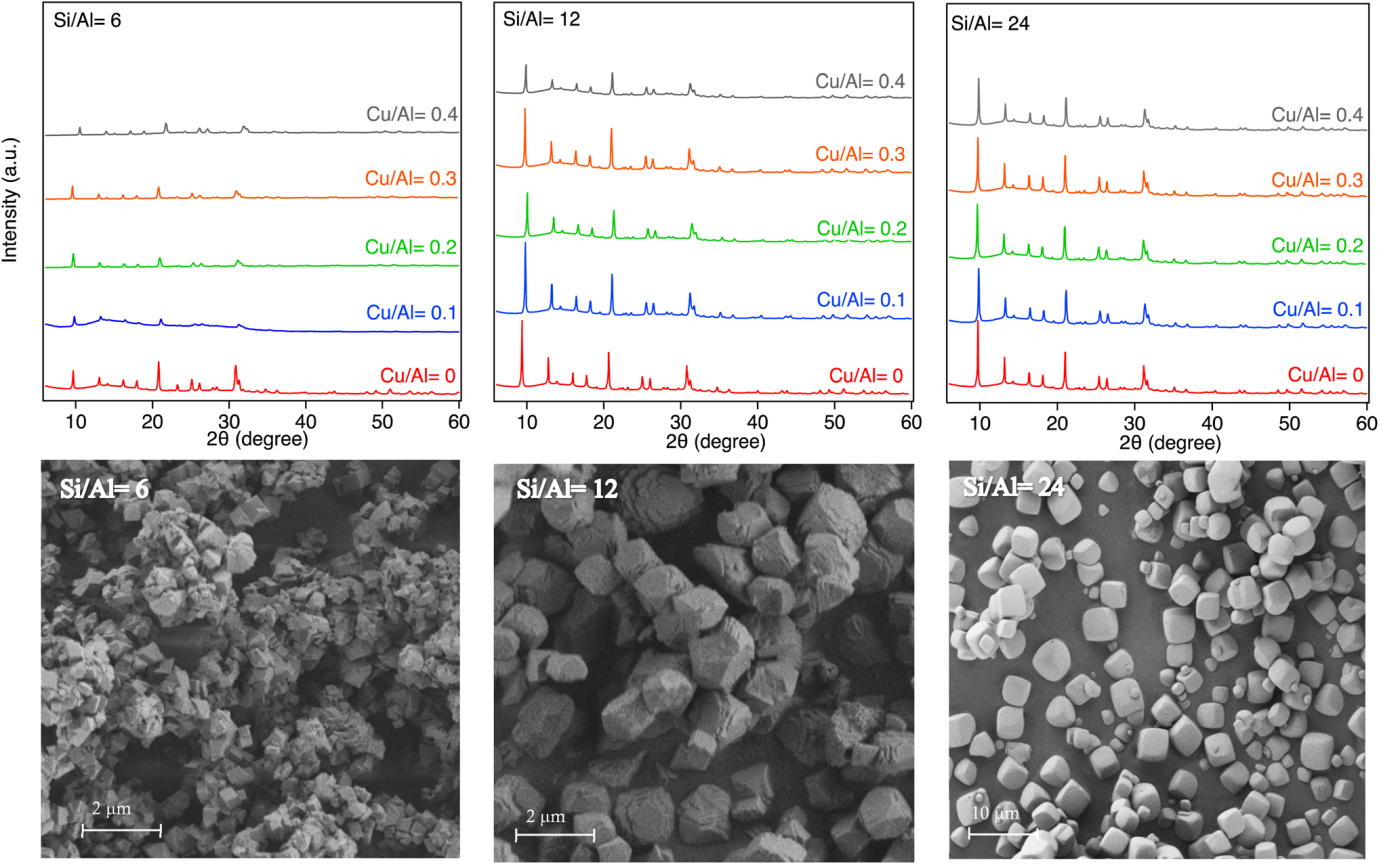
X-ray diffractograms of the samples with different Si/Al ratios (6, 12, and 24) and Cu contents, and SEM images for H-SSZ-13 samples (Cu/Al = 0).

The X-ray diffractograms for the samples before (H-form) and after Cu-exchange (Fig. 1) show diffraction peaks characteristic for SSZ-13 (CHA) at 2 theta = 9.5, 14.0, 16.1, 17.8, 20.7, 25.0, and 30.7°^22^ (JCPDS no; 52-0784).^42^ There were no obvious variations in the peaks' position for the different samples, which indicates that the crystal phase is not affected by changing Si/Al ratio or Cu-exchange.^39^ Based on the physical characterization of the samples, there are no indication of (crystalline) extra framework alumina. Moreover, there are no characteristic diffraction peaks that correlate with CuO phases (2 theta = 35.6 and 38.7°)^1^ in the Cu-exchanged samples. It can thus be concluded that the Cu species are highly dispersed or the formed CuO_*x*_ clusters are below the detection limit of the XRD analysis. It is noticeable that the diffraction peaks of the sample with Si/Al ratio = 24 have higher intensity. The lower peak intensity for Si/Al ratio = 6 is most likely due to the smaller crystals in this sample.^1,10,21^ The SEM images of the SSZ-13 samples, Fig. 1, show uniform cubic shapes characteristic for the CHA-framework,^21,39^ which is in agreement with the XRD results. Furthermore, the Si/Al = 24 has larger crystal size compared to the other samples. The specific surface area and specific pore volume for the samples are presented in Table S1. The specific surface area for the Si/Al = 6 samples decreases with Cu-exchange, for the other two Si/Al ratios there are only minor differences. The Si, Al, and Cu contents expressed as the Si/Al and Cu/Al molar ratio in Table S1 were obtained from elemental analysis (ICP-SFMS) and the results agree with the target values.

### 3.2. Ammonia sorption

#### 3.2.1. NH_3_ adsorption–desorption

The NH_3_ adsorption–desorption experiments were used to characterize the interaction between the NH_3_ and the SSZ-13 samples with varying Si/Al and Cu/Al molar ratios. These experiments provide a qualitative and quantitative analysis of various acid sites in the samples. To ensure similar starting conditions, all Cu samples were pretreated to contain mainly [Cu^I^(NH_3_)_2_]^+^ complexes, which upon increased temperature decompose to framework bound Cu^I^.

Generally, two main types of adsorbed NH_3_ species can be distinguished by DRIFT spectroscopy. One type is NH_3_ interacting with Brønsted acid sites, with the formation of NH_4_^+^ ions together with weakly adsorbed NH_3_ on NH_4_^+^ (NH_3_·NH_4_^+^ associations), giving rise to N–H bending vibration peaks in the 1500–1350 cm^−1^ range. The other type is NH_3_ coordinated to Lewis acid sites (Cu sites, extra framework Al^3+^) that commonly appear as an absorption band at around 1624 cm^−1^.^16^ Two configurations could exist for the Lewis acid sites; either [NH_3_–Cu–NH_3_]^+^, where copper forms a mobile complex, or an O_fw_–Cu–NH_3_ structure, where copper is coordinated to one framework oxygen (O_fw_).

**Fig. 2:**
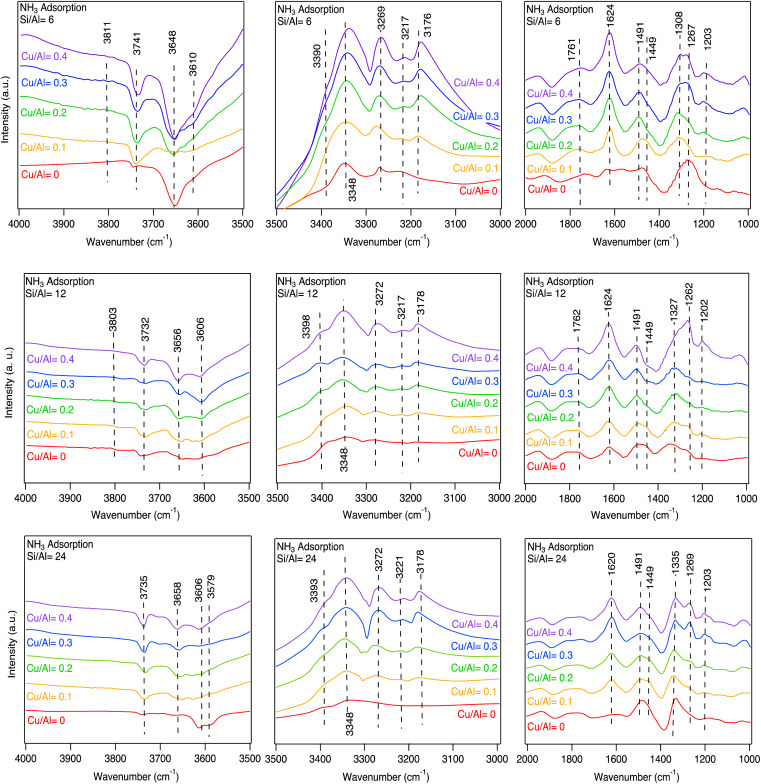
The *in situ* DRIFT spectra for NH_3_ adsorbed on different acid sites of the Cu-SSZ-13 samples with Si/Al = 6, 12, and 24 and different Cu/Al = 0, 0.1, 0.2, 0.3, 0.4 molar ratios at 70 °C (NH_3_ = 400 ppm in Ar).

DRIFT spectra recorded during the adsorption phase of the NH_3_ adsorption–desorption experiment are shown in Fig. 2. In the 4000–3500 cm^−1^ wavenumber region (left column), there are several negative bands around 3735, 3650 and 3606 cm^−1^, which are attributed to the attenuation of O–H stretching vibrations upon ammonia exposure. The band around 3735 cm^−1^ is related to adsorbed NH_3_ on Si–OH sites (silanol groups).^4,46^ The band around 3650 cm^−1^ can be assigned to OH on extra framework aluminium or ZCu–OH^27^ and the band around 3606 cm^−1^, corresponds to NH_3_ on Al–OH–Si (Brønsted sites).^3,16,17,21^ In the 3500–3000 cm^−1^ region (middle column) positive bands arises that are correlated to N–H stretching vibrations related to adsorbed NH_3_ and NH_4_^+^. Finally, the right column (2000–1000 cm^−1^) shows the corresponding N–H bending vibrations.^45^

**Fig. 3:**
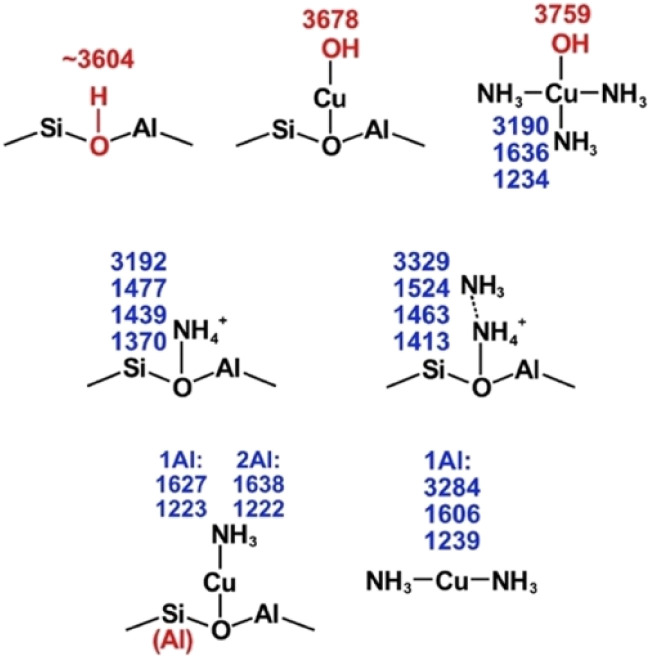
Calculated vibrations for stretching vibrations of O–H (red) and stretching and bending vibrations of N–H (blue) of potential species.

**Fig. 4:**
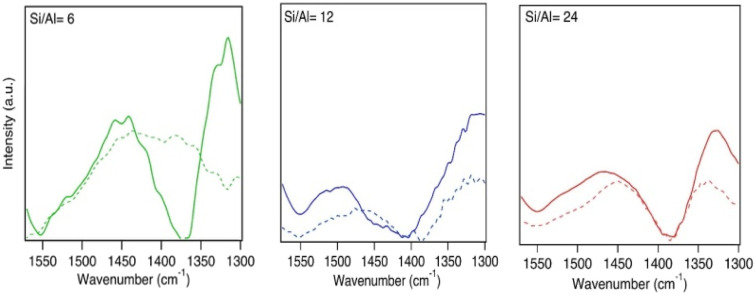
Simulated IR spectra for Cu^II^(NH_3_)_4_, Cu^I^(NH_3_)_2_, NH_4_^+^, and NH_4_^+^·NH_3_.

DFT calculations were performed to further elucidate the origin of the species during NH_3_ adsorption and NH_3_-TPD processes over Cu-SSZ-13 catalysts with different Si/Al and Cu/Al molar ratios. Fig. 3 shows sketches of the calculated structures and wavenumbers of relevant vibrations of potential species. For a further visual comparison with the infrared spectra, the simulated infrared intensities of key species were extracted and broadened by Gaussians as shown in Fig. 4.

For the Cu-free samples, experimental features in the region 3735 to 3579 cm^−1^ appear and are related to O–H stretching vibrations. According to the DFT results, Brønsted acid sites dominate before NH_3_ adsorption and the simulated stretching vibrations for O–H on different CHA sites are in the range from 3732 to 3604 cm^−1^ depending on the position of the OH-group.^3,46^ NH_3_, NH_4_^+^ and NH_4_^+^·NH_3_ ions form during the adsorption of NH_3_ over the Brønsted acid sites. The DRIFT results show two peaks in the 1491 to 1449 cm^−1^ region. The simulated spectra are dominated by the bending vibrations of 1477 and 1524 cm^−1^, respectively.

In the 3500–3000 cm^−1^ region, DRIFT spectra show the corresponding N–H stretching vibration at 3348 cm^−1^. The difference before and after NH_3_ adsorption is consistent with the negative peak around 3648 cm^−1^, which is plausibly related to O–H stretching vibrations from extra framework aluminium^27^ since the peak at 1491 cm^−1^ is more pronounced, NH_4_^+^·NH_3_ ions should dominate during NH_3_ adsorption conditions. With higher Si/Al, the number of Brønsted acid sites decreases, which gives rise to a smaller negative peak in the O–H stretching region.

Although the simulated spectra (Fig. 4) primarily emphasize N–H vibrations, the calculated O–H stretching modes (Fig. 3) span the 3600–3750 cm^−1^ region and correspond well to experimentally observed negative bands. The absence of explicit intensity inversion in the simulated spectra arises from the fact that DFT calculations describe isolated species, whereas the experimental negative features originate from depletion of OH groups upon NH_3_ adsorption. Nevertheless, the calculated frequencies support the assignment of bands at ∼3735 cm^−1^ to silanol groups and ∼3650 cm^−1^ to Cu–OH or extra-framework Al–OH species.

Considering the Cu containing samples, the catalysts are pretreated to obtain Cu^+^ and [Cu–OH]^+^ species onto which NH_3_ adsorbs. Starting with the peaks at around 3735 and 3650 cm^−1^ (Fig. 2) that become increasingly more negative with increasing Cu loading for the three-sample series. These peaks are likely attributed to O–H stretching vibrations on Cu bound to Brønsted sites. This attribution is supported by DFT calculations with the calculated peak at 3678 cm^−1^ (Fig. 3). The increasingly negative intensity is likely related to solvation of Cu by NH_3_. The DFT calculations show two possible ammonia solvated Cu species, *i.e.* Cu^I^(NH_3_)_2_ and Cu^II^(NH_3_)_3_OH. The stronger adsorption of NH_3_ on Cu species compared to Brønsted acid sites is also supported by DFT-calculated adsorption energies (see section 3.3), which explains the persistence of Cu–NH_3_ species at higher temperatures. Most probably the Cu^I^(NH_3_)_2_ complex is formed to a larger extent, hence resulting in negative contribution to the calculated peak at 3759 cm^−1^ that corresponds to OH stretching vibrations from ammonia solvated Cu. Furthermore, the DRIFT spectra show peaks at around 3272 and 3178 cm^−1^ that increases with increasing Cu content. These peaks are correlated with the simulated N–H stretching vibrations at 3284 and 3190 cm^−1^ for the Cu^I^(NH_3_)_2_ and Cu^II^(NH_3_)_3_OH species, respectively. The corresponding N–H bending vibrations are found in the DRIFT spectra at around 1624 and 1255 cm^−1^, which is supported by the calculated bending vibrations at 1636 and 1234 cm^−1^ for the Cu^II^(NH_3_)_3_OH species, and 1606 and 1239 cm^−1^ for Cu^I^(NH_3_)_2_.

By comparing the simulated infrared spectra for the different species with the measured spectra, we conclude that the experiments match the simulated infrared spectra of Cu^II^(OH)(NH_3_)_3_, Cu^I^(NH_3_)_2_, NH_4_^+^ and NH_4_^+^·NH_3_.

### 3.3. Temperature-programmed desorption of NH_3_

**Fig. 5:**
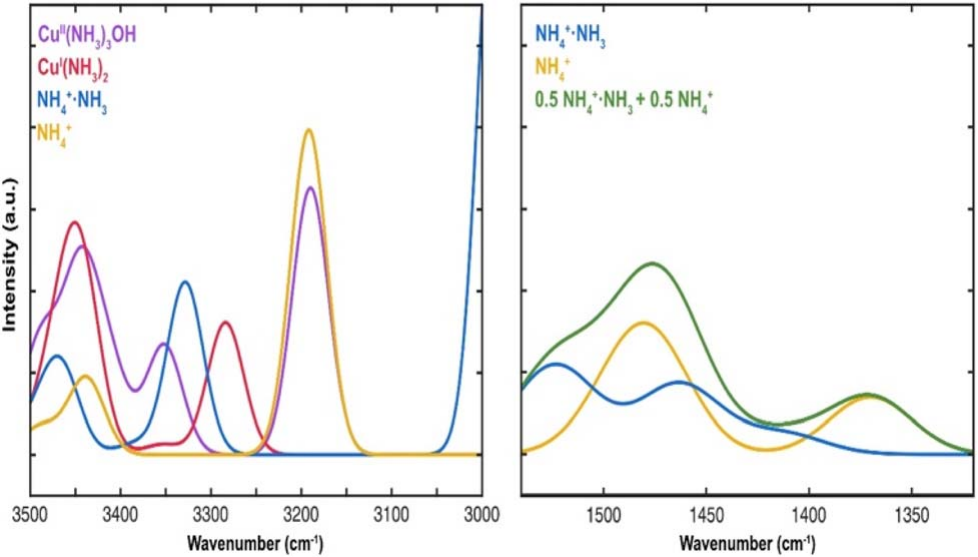
The *δ*(NH) spectra of NH_4_^+^ ions at desorption temperatures of 130 °C (solid line) and 200 °C (dashed line) during NH_3_-TPD over Cu-SSZ-13 samples with Si/Al = 6, 12, and 24 and Cu/Al = 0.4 molar ratios.

To further elaborate on the interpretation of the assignment of the surface species after NH_3_ exposure, ammonia TPD experiments were performed for all samples. The TPD profiles, including deconvolution with three Gaussian functions for each profile, are shown in Fig. 5. To focus on the main trends, we choose to make the deconvolution with peaks for three major desorption regions. These were allowed to vary in temperature and intensity for best total fit. For all samples the sum of the deconvoluted peaks shows reasonable correspondence with the TPD profile. The peak maxima of the deconvoluted peaks are located at 150–250 °C (low-temperature peak, LT), 250–350 °C (medium-temperature peak, MT), and 350–450 °C (high-temperature peak, HT).

The LT peak can be attributed to weakly adsorbed NH_3_ species on weak Lewis acid sites or physisorbed NH_3_ on the surface of the zeolite framework.^25,44^ The MT peak is attributed to NH_3_ on acid sites with medium strength, *e.g.* Lewis acid sites, and the HT peak is related to NH_3_ strongly bonded to Brønsted acid sites. The experimental TPD profiles generally increase in intensity with increasing Cu loading and decrease with increasing Si/Al molar ratio. These results are in line with NH_3_ adsorbed on Cu, which is clearly seen for the intensity of the LT and MT peaks. Each Cu^2+^-site can accommodate up to four ammonia species and Cu^+^-sites can accommodate up to two.^20^ Clemens *et al.*^7^ and Leistner *et al.*^32^ reported the same observations and they assigned both peaks (LT and MT) to various unspecified Cu species.

These findings indicate that for the SSZ-13 samples without Cu, NH_4_^+^·NH_3_ is the dominant species after NH_3_ adsorption at 70 °C, which contributes to the measured peak at 3348 cm^−1^ and decreases in intensity as the Si/Al molar ratio increases. The simulated IR spectra shows that when NH_4_^+^ exists, a peak of 3192 cm^−1^ would be present. The related peaks in the measured DRIFT spectra for the samples without Cu (3372 and 1449 cm^−1^) are weak but visible and show that NH_4_^+^ is present under NH_3_ adsorption conditions. For the Cu-SSZ-13 samples, Cu^I^ and [Cu(OH)]^+^ are the most stable species and therefore, forming Cu^I^(NH_3_)_2_ and Cu^II^(OH)(NH_3_)_3_ after NH_3_ adsorption. The simulations show that Cu^I^(NH_3_)_2_ and Cu^II^(OH)(NH_3_)_3_ species give rise to peaks at 3284 cm^−1^ and 3190 cm^−1^ (red and purple curves in Fig. 4), respectively. It is obvious from Fig. 2 that the peaks around 3269 cm^−1^ and 3176 cm^−1^ correlate positively with the Cu loading, but negatively with the Si/Al ratio where it slightly shifts to higher wavenumbers with increasing Si/Al molar ratio. This is consistent with the calculations, confirming that the amount of Cu^I^(NH_3_)_2_ and Cu^II^(OH)(NH_3_)_3_ increases with decreasing Si/Al molar ratio and increasing Cu loading.

**Fig. 6:**
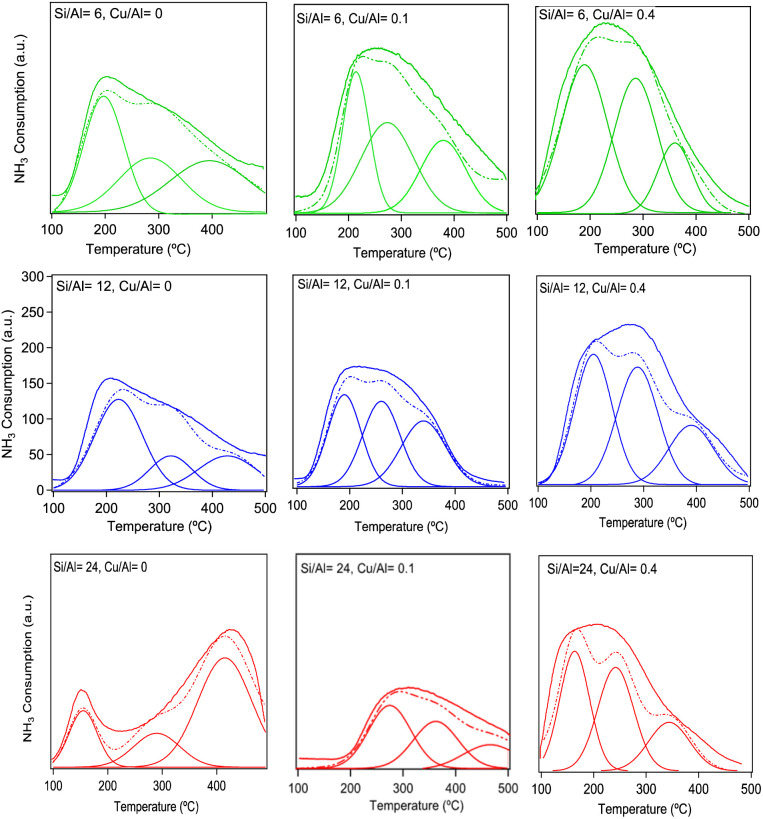
The *δ*(NH) spectra of NH_4_^+^ ions at desorption temperatures of 130 °C (solid line) and 200 °C (dashed line) during NH_3_-TPD over Cu-SSZ-13 samples with Si/Al = 6, 12, and 24 and Cu/Al = 0, 0.1, and 0.4 molar ratios.

As can be noted from Fig. 6, temperature has a significant effect on the corresponding spectra of NH_4_^+^ ions. The adsorption energy of NH_3_ on NH_4_^+^·NH_3_ is −0.89 eV, which indicates that NH_4_^+^·NH_3_ should start to decompose into NH_4_^+^ at higher temperatures and, thus, lead to a shift of the peaks. To further relate the experimental TPD features to the nature of the adsorption sites, we also consider NH_3_ adsorption on Cu species. DFT calculations indicate that NH_3_ binds more strongly to Cu sites than to NH_4_^+^, with adsorption energies typically in the range of approximately −1.0 to −1.3 eV for Cu^+^ and −1.2 to −1.5 eV for Cu^2+^ species. The stronger interaction of NH_3_ with Cu species compared to NH_4_^+^ is consistent with the assignment of the medium-temperature (MT) desorption peak to NH_3_ coordinated to Cu sites, while NH_4_^+^-related species dominate the high-temperature (HT) region.

This comparison establishes a direct link between the calculated adsorption energetics and the experimentally observed desorption temperatures, thereby supporting the interpretation of the TPD profiles.

At low temperature (130 °C in Fig. 6), both NH_4_^+^ and NH_4_^+^·NH_3_ may exist and gives the (0.5 NH_4_^+^ + 0.5 NH_4_^+^·NH_3_) curve in Fig. 4, which has a broad signature between 1500 to 1540 cm^−1^ and a main peak at 1480 cm^−1^. With increasing temperature, the weaker adsorbed NH_3_ on NH_4_^+^·NH_3_ will desorb, and the spectra will change more to the shape of NH_4_^+^. One obvious change is that as the amount of NH_4_^+^·NH_3_ decreases, the intensity of the peak at 1524 cm^−1^ also becomes weaker. From the experimental measurements in Fig. 6, the leftmost shoulder of each spectrum becomes lower at higher temperature, which is consistent with the simulations. Comparing the TPD results in Fig. 5 with the ammonia-desorption spectra in Fig. 6, it can be observed that the NH_4_^+^·NH_3_ peak (broad signature between 1540 and 1500 cm^−1^) decomposes at relatively low temperatures, *i.e.* in the LT-region. The remaining NH_4_^+^ species is stable until decomposition in the HT-region.

To further clarify the distribution of peak contributions, a rough quantitative analysis of the deconvoluted profiles was performed to estimate the relative contributions of low-, medium-, and high-temperature peaks. Although exact numerical integration was not available, visual assessment of the fitted peak areas allows a reasonable comparison across the samples.

For Si/Al = 6, the low-temperature contribution decreases from ∼35% (Cu/Al = 0) to ∼20% (Cu/Al = 0.4), while the medium-temperature region increases from ∼40 to ∼50%, and the high-temperature contribution rises slightly from ∼25 to ∼30%.

For Si/Al = 12, a similar trend is observed, with the low-temperature contribution decreasing from ∼30 to ∼20%, the medium-temperature region remaining dominant at ∼45–50%, and the high-temperature contribution increasing more significantly from ∼25 to ∼35% at higher Cu loading.

For Si/Al = 24, the distribution shows a slight deviation at intermediate Cu loading, where the low-temperature contribution increases to ∼35% at Cu/Al = 0.1, before decreasing again to ∼20% at Cu/Al = 0.4. Meanwhile, the high-temperature contribution increases from ∼25 to ∼35%, indicating a shift toward stronger adsorption sites at higher Cu content.

Overall, increasing Cu/Al molar ratio leads to a systematic shift from low-temperature to higher-temperature peak contributions, suggesting an increase in stronger adsorption or binding sites. The medium-temperature peaks remain the dominant contribution across all samples, while the high-temperature peaks become increasingly significant at higher Cu loadings.

## 4. Conclusions

The aim of the study was to characterize the nature of ammonia storage sites in H-SSZ-13 and Cu-SSZ-13 with different Si/Al and Cu/Al molar ratios. All samples were successfully synthesized according to the XRD results showing the CHA framework structure, however, the sample with Si/Al molar ratio of 6 most likely contain extra framework alumina. Copper was successfully incorporated in the samples in the range Cu/Al molar ratio of 0.1–0.4 using incipient wetness impregnation of copper nitrate solution. Ammonia sorption DRIFTS experiments over Cu-free samples show that NH_3_, NH_4_^+^ and NH_4_^+^·NH_3_ ions form during the adsorption of NH_3_ over the Brønsted acid sites at 70 °C. For the Cu-containing samples, the catalysts were pretreated to form Cu^I^ species. DRIFT results show that peaks at around 3735 and 3650 cm^−1^ become increasingly more negative with increasing Cu loading, which is likely related to solvation of Cu by NH_3_. DFT calculations show two possible ammonia solvated Cu species, *i.e.* Cu^I^(NH_3_)_2_ and Cu^II^(NH_3_)_3_OH. By comparing the simulated infrared spectra for the different species with the measured spectra, we conclude that the experiments match the simulated infrared spectra of Cu^II^(OH)(NH_3_)_3_, Cu^I^(NH_3_)_2_, NH_4_^+^ and NH_4_^+^·NH_3_. The results reveal that the balance between Brønsted and Cu-related NH_3_ storage sites is governed by both the Si/Al ratio and the Cu/Al ratio, leading to distinct adsorption regimes that cannot be inferred from Cu loading alone.

Furthermore, the TPD results show that the NH_4_^+^·NH_3_ peak decomposes at relatively low temperatures, *i.e.* in the low-temperature region. The remaining NH_4_^+^ species is stable until decomposition in the high-temperature region. The combined experimental and DFT results demonstrate that the stronger adsorption of NH_3_ on Cu sites compared to NH_4_^+^ governs the temperature-dependent desorption behavior.

The present study provides a unified molecular-level interpretation of NH_3_ storage in Cu-SSZ-13 by directly linking DRIFTS features, NH_3_-TPD desorption regions, and DFT-identified species. Importantly, the results demonstrate that the interplay between Si/Al ratio and Cu loading governs the distribution of NH_3_ storage sites, revealing trends that cannot be deduced from varying a single parameter alone. This combined experimental–theoretical framework provides new insight into the origin of LT, MT, and HT desorption features and their relation to specific Cu–NH_3_ and Brønsted-bound species.

## Author contributions

Ghodsieh Isapour: investigation– conducting a research and investigation process, specifically performing the experiments, or data/evidence collection. visualization–preparation, creation and/or presentation of the published work, specifically visualization/data presentation. Writing the original draft– preparation, creation and/or presentation of the published work, specifically writing the initial draft (including substantive translation). Yingxin Feng: formal analysis – application of statistical, mathematical, computational, or other formal techniques to analyze or synthesize study data. Writing the original draft– writing the initial draft for DFT calculation section. Henrik Grönbeck: conceptualization – ideas; formulation or evolution of overarching research goals and aims. Magnus Skoglundh: conceptualization– ideas; formulation or evolution of overarching research goals and aims. supervision – oversight and leadership responsibility for the research activity planning and execution, including mentorship external to the core team. Hanna Härelind: conceptualization– ideas; formulation or evolution of overarching research goals and aims. Supervision – oversight and leadership responsibility for the research activity planning and execution, including mentorship external to the core team.

## Conflicts of interest

There are no conflicts of interest to declare.

## Supplementary Material

RA-016-D6RA03025D-s001

## Data Availability

Additional data supporting the findings of this study are available from the corresponding author upon reasonable request. Supplementary information (SI): the datasets generated and analyzed during the current study are available within the article and its electronic supplementary information. See DOI: https://doi.org/10.1039/d6ra03025d.
