# Supplementary material for: Nature of ammonia storage sites in H-SSZ-13 and Cu-SSZ-13
Source: RSC Adv. 2026 May 26;16(31):28182–90. doi: 10.1039/d6ra03025d (PMC13213573; doi:10.1039/d6ra03025d)
Supplement: RA-016-D6RA03025D-s001 [file RA-016-D6RA03025D-s001.pdf]

Supplementary Information (SI) for RSC Advances.
This journal is © The Royal Society of Chemistry 2026

Supporting Information

Nature of ammonia storage sites in H-SSZ-13 and Cu-SSZ-13

Ghodsieh Isapour[a]*, Yingxin Feng[b], Henrik Grönbeck[b], Magnus Skoglundh[a] and Hanna Härelind[a]*

Competence Centre for Catalysis, Chalmers University of Technology, Gothenburg, Sweden, , SE-41296, Gothenburg, Sweden

[a]*Department of Chemistry and Chemical engineering, Division of Applied Chemistry*

[b]*Department of Physics, Division of Chemical Physics*

Available Supporting Information

Table S1. Si/Al ratio, Cu/Al ratio, specific surface area, and specific pore volume of the samples.

Sample		Specific surface area ($m^2.g^{-1}$)	Specific pore volume ($cm^3.g^{-1}$)	Si/Al molar ratio	Cu/Al molar ratio
Si/Al	Cu/Al				
6	0	603	0.01	4.8	-
6	0.1	486	0.07	4.6	0.11
6	0.2	499	0.19	4.6	0.18
6	0.3	475	0.03	5.1	0.33
6	0.4	482	0.19	5.7	0.43
12	0	650	0.3	12.6	-
12	0.1	670	0.28	12.6	0.1
12	0.2	659	0.27	13.5	0.22
12	0.3	649	0.27	14	0.33
12	0.4	631	0.26	14	0.36
24	0	613	0.25	25	-
24	0.1	613	0.29	22.2	0.08
24	0.2	638	0.28	25.4	0.19
24	0.3	644	0.29	26.12	0.25
24	0.4	699	0.25	24.5	0.4
